# Understanding the role of bitter taste perception in coffee, tea and alcohol consumption through Mendelian randomization

**DOI:** 10.1038/s41598-018-34713-z

**Published:** 2018-11-15

**Authors:** Jue-Sheng Ong, Daniel Liang-Dar Hwang, Victor W. Zhong, Jiyuan An, Puya Gharahkhani, Paul A. S. Breslin, Margaret J. Wright, Deborah A. Lawlor, John Whitfield, Stuart MacGregor, Nicholas G. Martin, Marilyn C. Cornelis

**Affiliations:** 1 QIMR Berghofer Medical Research Institute, Brisbane, Australia; 2 School of Medicine, University of Queensland, Brisbane, Australia; 3 University of Queensland Diamantina Institute, University of Queensland, Brisbane, Australia; 4 Department of Preventive Medicine, Northwestern University Feinberg School of Medicine, Chicago, IL USA; 5 Monell Chemical Senses Center, Philadelphia, PA 19104 USA; 6 Department of Nutritional Sciences, School of Environmental and Biological Sciences, Rutgers University, New Brunswick, NJ 08901 USA; 7 Queensland Brain Institute, University of Queensland, Brisbane, Queensland 4072 Australia; 8 Centre for Advanced Imaging, University of Queensland, Brisbane, Queensland 4072 Australia; 9 MRC Integrative Epidemiology Unit at the University of Bristol, Oakfield House, Oakfield Grove, Bristol, BS8 2BN UK; 10 Population Health Science, Bristol Medical School, University of Bristol, Canynge Hall, Whiteladies Road, Bristol, BS8 2PS UK

## Abstract

Consumption of coffee, tea and alcohol might be shaped by individual differences in bitter taste perception but inconsistent observational findings provide little insight regarding causality. We conducted Mendelian randomization analyses using genetic variants associated with the perception of bitter substances (rs1726866 for propylthiouracil [PROP], rs10772420 for quinine and rs2597979 for caffeine) to evaluate the intake of coffee, tea and alcohol among up to 438,870 UK Biobank participants. A standard deviation (SD) higher in genetically predicted bitterness of caffeine was associated with increased coffee intake (0.146 [95%CI: 0.103, 0.189] cups/day), whereas a SD higher in those of PROP and quinine was associated with decreased coffee intake (−0.021 [−0.031, −0.011] and −0.081 [−0.108, −0.054] cups/day respectively). Higher caffeine perception was also associated with increased risk of being a heavy (>4 cups/day) coffee drinker (OR 1.207 [1.126, 1.294]). Opposite pattern of associations was observed for tea possibly due to the inverse relationship between both beverages. Alcohol intake was only negatively associated with PROP perception (−0.141 [−1.88, −0.94] times/month per SD increase in PROP bitterness). Our results reveal that bitter perception is causally associated with intake of coffee, tea and alcohol, suggesting a role of bitter taste in the development of bitter beverage consumption.

## Introduction

Coffee, tea and alcohol are widely consumed beverages with bitter taste^1^ and have been implicated in both beneficial and adverse health effects^2,3^. Individual differences in metabolizing caffeine^4,5^ and ethanol^6–10^ present in these beverages determine their consumption level, whereas the influence of taste factors remains unclear.

The relationship between the perception of bitter compounds, such as propylthiouracil (PROP), quinine, and caffeine, and the consumption level of these bitter beverages have been inconsistent across observational studies^11–22^. As performing taste tests is a resource-intensive process, these investigations likely lacked sufficient power to convincingly rule out moderate effects. Potential confounders or illnesses that both affect taste perception and consumption in these studies limits their ability to provide unbiased causal effects. Fortunately, these issues can be overcome by recent advances in taste genetics and statistical methodology.

Heritability for the perceived intensity of PROP, quinine, and caffeine have been estimated to be 0.73, 0.40, and 0.36 in classical twin studies^23,24^. Furthermore, candidate gene and genome-wide association studies (GWAS) have pinpointed precise genetic factors^25–27^, including SNPs within the bitter taste receptor gene *TAS2R38* for PROP and SNPs within the cluster of bitter taste receptors genes on chromosome 12 for quinine and caffeine. Identification of these genetic variants provides an opportunity to apply Mendelian randomization (MR), a technique commonly used in disease epidemiology, to test the causal relationship between bitter taste and an outcome of interest using genetic variants (e.g. SNPs) as instrumental variables (IVs). The fundamental principle of MR is based on Mendel’s Law of Independent Assortment whereby genetic variants are shuffled at meiosis, conceptually mimicking a “natural” randomized trial^28–30^. Since genetic variants are randomized at conception, they can be used as risk factor instrumental variables that are generally free from confounding and reverse causality to evaluate the relationship of a risk factor on a specific outcome of interest^31,32^.

In this study, we investigate the causal relationship between bitter taste perception and the consumption of coffee, tea and alcohol through a MR framework. We use confirmed genetic markers for the perception of PROP (rs1726866), quinine (rs10772420) and caffeine (rs2597979) separately as genetic proxies for bitter taste perception and test their associations with the consumption of coffee, tea and alcohol among more than 400,000 participants in the UK Biobank cohort^33^.

## Methods

### Study design

We employed a two-sample Mendelian randomization design^30^, wherein the IV-taste perception association (ratio denominator) and IV-consumption association (ratio numerator) were obtained from two independent sample sets. The SNP-taste perception estimates were derived from a GWAS of taste perception^27^ described below (sample 1), while the SNP-beverage consumption association was examined in the UK Biobank (sample 2)^33^.

### Genetic instruments for bitter taste (sample 1)

Genetic instruments for perceived intensity of PROP, quinine and caffeine were obtained from the largest GWAS of these traits to-date^27^, which included 1757 twins and their siblings (aged 12–25; 54% females; all European ancestry) from a subset of the Brisbane Adolescent Twin study^34^. All three taste-associated SNPs were genome-wide significant (rs1726866 in *TAS2R38* on chromosome 7 accounted for approximately 46% variance in PROP (P = 5.6e-198); rs10772420 and rs2597979 in the cluster of bitter taste receptor genes on chromosome 12 respectively accounted for 6% of the variance in quinine (P = 7.8e-23) and 2% of the variance in caffeine (P = 4.2e-8)) and replicated findings from previous smaller GWAS^25,26,35^. The corresponding genetic instrument F-statistics were 901.1 (PROP), 96.8 (Quinine) and 30.0 (Caffeine) respectively, indicating all of the SNPs used were strong instruments (Supplementary Table 1). In the GWAS, taste intensity ratings were square root transformed and then converted into standard deviation units. Here, we assigned the effect allele (EA) to represent the allele associated with higher intensity ratings on bitterness. The EA for rs1726866 (G allele), rs10772420 (A allele), and rs2597979 (G allele) increased the intensity rating of PROP, quinine, and caffeine by 0.965, 0.337, and 0.264 standard deviations (SD), respectively.

### UK Biobank (sample 2)

The UK Biobank recruited 502,650 participants (5% of those invited) aged 37–73 years at 21 centres across England, Wales and Scotland in 2006–2010^33^. Written informed consent was obtained from participants and the study has been approved by the UK National Health Service National Research Ethics Service. Full details of participant recruitment and data collection have been previously published^36^ as have details of the genome-wide data collection and its quality control^33^.

### UK Biobank Genetic data

All UK Biobank participants have been genotyped using the Affymetrix UK BiLEVE Axiom array or Affymetrix UK Biobank Axiom® array comprising 805,426 markers in the official release. Imputations were performed using IMPUTE2 and UK10K haplotype and Haplotype Reference Consortium (HRC) reference panels, as described elsewhere^33^. For the current analysis, the three SNPs of interest were genotyped (rs1726866, rs10772420) or imputed (rs2597979) with high quality (INFO score >0.99) based on the HRC reference panel samples. Our present analyses focused on the population of white-British ancestry, as determined by similarity of ancestral principal component values (PC1, PC2) to those who self-reported and were classified as white-British^37^. Using this criterion, we identified 438,870 individuals of white-British ancestry.

### UK Biobank Coffee, tea and alcohol consumption

Self-reported data on tea, coffee and alcohol consumption were collected using the touch-screen questionnaire completed by UK Biobank participants at the assessment centre. Amounts of coffee and tea consumption were quantified separately e.g. “How many cups of coffee do you drink each day? (including decaffeinated coffee)” and “How many cups of tea do you drink each day? (including black and green tea)”. For alcohol, participants were asked “About how often do you drink alcohol?” and to report on their drinking behaviour on a 6-point frequency scale (Never, Special occasions only, 1–3 times a month, 1–2 times a week, 3-4 times a week, Daily or almost daily). Participants who preferred not to answer were excluded from the analyses. We converted the alcohol intake scores into monthly frequencies (frequency of alcohol consumption per month) from the 6-point frequency scale to 0, 1, 2, 6, 15, 30 times per month. For participants completing the assessment at multiple times, we computed their average consumption.

To assess risks of heavy-consumption, the cut-off for light/non-drinkers and heavy drinkers are set at 20th and 80th percentile of the consumption distribution. For coffee, light/non-drinkers were classified as participants with <2 cups per day and heavy drinkers as >4 cups per day of coffee intake. The cut-offs were <2 cups/day and >5 cups/day for tea-drinking. For alcohol, non-drinkers were defined as individuals reporting no consumption of alcohol, while heavy-drinkers were individuals who consume more frequently than 3-4 times weekly.

### Statistical Analysis

Association between each bitter-taste SNP and bitter beverage consumption were estimated through linear and logistic additive models using BOLT-LMM^38^ (linear and logistic) and again with *plink2*^39^ (logistic). Covariates included age, sex and the first 10 principal components (PC). The use of BOLT-LMM allows the use of a linear mixed model to adjust for cryptic relatedness, hence maximizing power as related individuals can be retained in the analyses. We reported estimates derived from BOLT-LMM for the main analyses. For binary traits, such as drinker status, we additionally compute the SNP-associations using software *plink2*^39^ after excluding related individuals (n ~ 36,000), with the results retained as sensitivity analyses.

The MR estimate of the effect of bitter taste perception on consumption was calculated using the wald-type ratio estimator^28,40^. The causal magnitude of association is estimated through ${\beta }_{wald}={\beta }_{bev}\,/{\beta }_{bitter}$, where *β*_*bev*_ refers to the magnitude of association on quantity of bitter beverages for each perception-increasing allele and *β*_*bitter*_ refers to the magnitude of association for per-SD change in square-rooted perception score for each perception-increasing allele. For the analyses of logistic traits (e.g. non- vs heavy-drinkers), the equivalent causal odds ratio were estimated using log $O{R}_{wald}=\,\mathrm{log}\,O{R}_{drinkstatus}/{\beta }_{bitter}$ where log *OR*_*drinkstatus*_ refers to the (log) increase in heavy-drinking risk per perception-decreasing allele. The standard errors of our MR estimates were derived using the delta method^41^. Estimates hence reflect the change in consumption quantity per SD change in bitter perception score in PROP, quinine and caffeine. All statistical analyses were performed using in-house scripts written in the statistical package R. As we investigated 3 different bitter stimuli against 3 different beverages of interest in our analyses, we conservatively set our Bonferroni-corrected P-value to be 0.00556 (0.05/9).

We further assessed evidence for SNP associations with potential confounders through a look-up effort on publicly available GWAS summary results. Databases such as the GENEATLAS UK Biobank GWAS repository^42^ and the PhenoScanner web interface^43^ were used. Here potential confounders are selected based on prior evidence of association with coffee intake or traits which might potentially associate coffee intake in a reverse causality fashion. These include smoking behaviour, socio-economic status, BMI, diet, sleep pattern and gastrointestinal disorders.

### Disclaimer

All authors did not attempt to de-identify participants from the UKB across the use of two UKB application data (performed separately).

## Results

Table 1 summarizes the baseline characteristics including self-reported measurements of bitter beverage consumption among 438,870 individuals that were included in our analyses. The genotype frequencies for each of the bitter SNPs are shown in Supplementary Table 2. Power calculation for our MR analyses reveal that we had very good power to detect even very small effect sizes (Supplementary Table 3).

**Table 1:** Baseline characteristics of 438,870 individuals from the UK Biobank cohort included in the analyses.

Variable	Statistics	Value based on 438, 870 participants
Age	Mean (SD)	56.5 (8.1)
Sex	N of males (%)	223,040 (45.8%)
Height (cm)	Mean (SD)	168.5 (9.3)
BMI	Mean (SD)	27.42 (4.8)
Smoking	N (%)	
Ever smoked		194,764 (40.2%)
Self-reported alcohol drinking frequency	N (%)	
Never		39,434 (8.1%)
Special occasions only		55,197 (11.4%)
1–3 times/month		56,299 (11.6%)
1–2 times/ week		123,195 (25.3%)
3–4 times/week		115,327 (23.7%)
Daily or almost daily		96,870 (19.9%)
Coffee consumption	Mean (SD)	
Coffee (cups per day)		2.13 (2.1)
Tea consumption	Mean (SD)	
Tea (cups per day)		3.51 (2.9)

BMI is the body mass index. N refers to number (Sample size). SD refers to standard deviation.

### Mendelian randomization estimates of bitter perception on coffee and tea consumption

**Figure 1:**
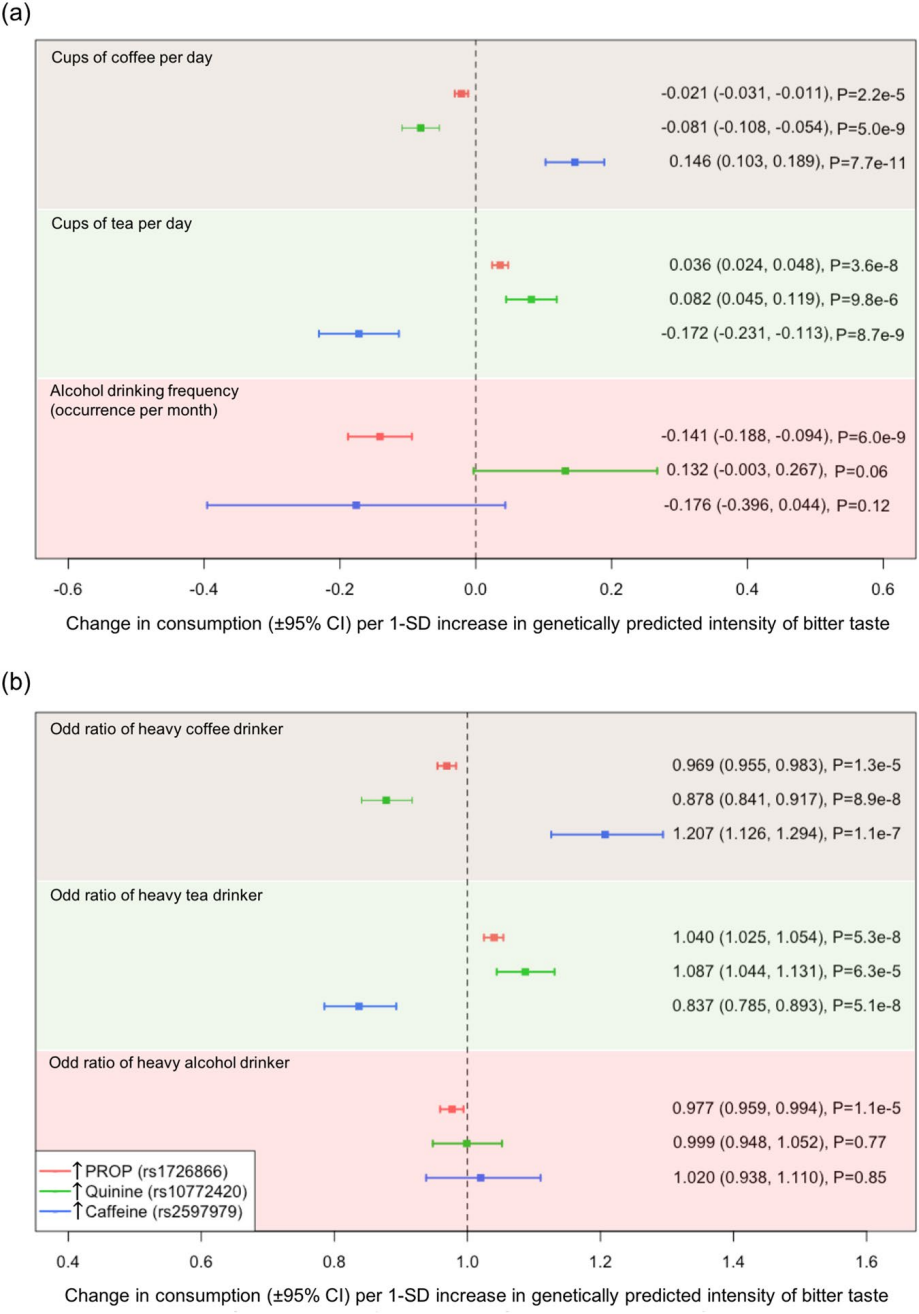
Causal effects of perceived bitterness of PROP, quinine, and caffeine on (**a**) the intake of coffee, tea, and alcohol and (**b**) the odds ratio of being a heavy coffee, tea, and alcohol drinker. The bar around the box represents the 95% confidence intervals around the point estimate.

Power calculation for our MR analyses indicated that we had very good power to detect even very small effect sizes on changes in consumption due to differences in bitter perception. Associations between SNPs associated with perception of bitterness and the consumption of bitter beverages are summarized in Supplementary Tables 4 (linear) and 5 (logistic). The approximation of SNP-beverage estimates derived from BOLT-LMM to log(OR) values can be found in Supplementary Table 5. Mendelian randomization causal effect estimates of tea/coffee/alcohol consumption per SD increase in perceived bitterness are shown in Fig. 1a. We identified robust associations between genetically predicted higher perception for each bitter taste and coffee consumption (P < 1e-4); however the effect sizes were relatively weak. The magnitude of association for higher caffeine perception (rs2597979) is the largest (beta = 0.039; 95% CI: 0.027,0.050) and was consistently in the opposite direction compared to perception of PROP (rs1726866) and quinine (rs10772420). Higher perception of caffeine is causally associated with increased risk of being a heavy coffee drinker (Causal OR = 1.207; 95% CI: 1.126, 1.294), see Fig. 1b), with similar evidence for association with higher cups per day, but higher perception of PROP and quinine results in lower coffee consumption.

Genetically predicted higher PROP and quinine intensity was associated with increased tea consumption, with a change of 0.034 (95% CI: 0.022,0.047) and 0.028 (95% CI: 0.015, 0.040) cups per day per SD increase in perception score. In contrast, higher caffeine perception leads to a decrease in tea consumption (beta = −0.045, 95% CI: −0.061, −0.030). Similar patterns of association were observed when comparing non-tea drinkers against heavy tea drinkers (see Fig. 1b).

### Conditional Mendelian randomization analyses to address inverse correlation between coffee and tea

Given the opposite pattern of results for coffee and tea, we investigated whether this was driven by the negative correlation between coffee and tea intake in the UK Biobank (r = −0.3). We performed conditional analyses by adjusting for cups per day of tea consumption when estimating the MR causal estimates on bitter perception of coffee, and vice versa. Our conditional analyses (Supplementary Table 6) revealed that the magnitude of association between predicted quinine and caffeine perception with coffee intake were stronger after conditioning for tea intake, as well as the association between predicted PROP and caffeine perception and tea intake after adjusting for coffee intake, although there is limited evidence for statistical heterogeneity between these and the original estimates.

When stratified by non-drinker status for tea (or coffee) for the association between bitter taste and coffee (or tea) (Supplementary Tables 7 and 8), the direction of effect was largely consistent with the unstratified model - with larger standard errors due to a loss of effective sample size. However, the magnitude of positive (negative) association for caffeine perception on coffee (tea) among non-tea (coffee) drinkers was larger than the unstratified model (e.g. beta_coffee among non-tea drinkers = 0.124; as compared to 0.039 from the original model), suggesting a potential beverage preference based on caffeine perception. Complete results for the conditional and stratified analyses can be found in Supplementary Tables 6–8.

### Mendelian randomization estimates for alcohol intake

The MR analysis of bitter taste perception on alcohol varied by stimuli (Fig. 1). For PROP, increased predicted intensity was associated with lower alcohol consumption (beta = −0.136 times/month (95% CI: −0.182, −0.090) for every SD increase in PROP intensity). Our MR analyses revealed weak evidence that genetically predicted increased quinine perception is associated with higher alcohol consumption as the confidence interval overlaps zero. Genetically predicted caffeine perception was weakly associated with alcohol consumption, with wide confidence intervals that included the null value (beta = −0.047, 95% CI: −0.105, 0.011). For drinker status, direction of association was largely consistent (Fig. 1b), but none of the associations between predicted bitter taste and drinking status were statistically significant at the conventional p-value of 0.05 after correcting for multiple testing. Through the stratified MR analyses by types of alcohol, we further showed the association between PROP and alcohol intake is mainly driven by red wine (Supplementary Table 9).

### Investigating sex differences on the causal association between bitter taste and bitter beverage consumption

In light of previously suggested interaction of sex on the association between bitter perception and these beverages^14,16,19^, we attempted to evaluate whether these differences were observed in our data, by performing our MR analyses separately for each sex (Supplementary Table 10). We found no consistent evidence to support a sex difference, except for the association between caffeine perception and tea intake (p = 0.007) which appeared to be much stronger in females (for every SD increase in caffeine perception score, beta = −0.261 cups per day (95% CI: −0.341, −0.182) in females and beta = −0.091 cups per day (95% CI: −0.184, 0.004) in males).

## Discussion

We investigated the effect of perception of different bitter compounds on tea, coffee and alcohol consumption in a large population-based cohort (UK Biobank) using a MR approach. Our findings showed that a higher perceived intensity of PROP and quinine decreased coffee consumption and that a higher perceived intensity of caffeine increased coffee consumption; opposite relationships were observed for tea consumption. For alcohol, a higher predicted intensity of PROP resulted in lower consumption but the perception of quinine and caffeine had no clear influence.

Increased predicted perceived intensity of PROP and quinine leads to a lower coffee intake and a lower risk of being a heavy coffee drinker. The direction of association could be attributed to the inborn aversion towards bitterness, which is a defensive mechanism that prevents ingestion of poisonous food^44^. Previous studies investigating the effect of PROP taster status on coffee consumption have reported null findings^12,13,17,18,22^ (except for one study of Polish elderly women (n = 118) showed that tasters consume coffee more frequently)^16^, but with sample sizes between 8 and 3328, these studies may have been underpowered. In the current analysis of over 438,870 cohort participants, the *TAS2R38* genotype corresponding to increased perceived PROP intensity was associated with a lower consumption of coffee. The effect size is notably small, thus warranting a large sample size to detect. For the association with the perception of quinine, our finding can be supported by an earlier report that coffee drinkers tend to be less sensitive to quinine^15^. The effect of quinine on coffee is stronger than that of PROP, presumably because the quinine naturally occurs in coffee^45^ whereas PROP does not.

While the perception of caffeine demonstrated the largest magnitude of association, its direction of effect was opposite to the other two bitter substances PROP and quinine, i.e. an increased predicted perceived intensity of caffeine leads to a higher intake of coffee. Caffeine is a non-volatile component of coffee and it contributes to the perceived strength, body and bitterness of coffee^46^, which has been related to its intake^11,15^. It is possible that coffee consumers acquire a taste for (or an ability to detect) caffeine given the learned positive reinforcement (i.e. stimulation) elicited by caffeine.

For the relationship between bitter taste perception and tea intake, we showed that a higher predicted perceived intensity of PROP and quinine increased tea intake and a higher predicted perceived intensity of caffeine decreased intake. These effects of bitter taste on tea consumption were in the opposite direction compared with those on coffee consumption, consistent with the negative correlation between coffee and tea intake in the UK Biobank cohort. A negative relationship of similar magnitude has also been reported in an Australian population (N = 3908)^47^. Nevertheless, our sensitivity analyses, which used the IV estimate on tea conditional on coffee (and vice versa), showed the same direction of association and revealed a robust association between tea intake and the perception of PROP and caffeine (and between coffee intake and the perception of quinine and caffeine).

For alcohol, our MR results indicated that increased predicted perceived intensity of PROP led to a lower intake of alcohol. It has been reported that alcohol elicits bitterness in humans^48,49^ and such bitter sensation can mediate consumption behaviour^50^. Findings from this study could be supported by previous observations that a higher perceived intensity of PROP is associated with a stronger perceived intensity of alcohol and a lower intake^14,19,50,51^. While inconsistencies have been reported between sexes^14,16,19^, here we found no strong evidence of a difference between males and females in the large UK Biobank cohort.

The strengths of our study include the use of a two-sample approach in MR to avoid biased estimates^40^. Given that each of these SNPs are functionally related to taste biology, each of the SNP used were strong instruments backed with statistical evidence as well as biological plausibility. Taste perception can change overtime, whereas MR analyses evaluate the impact of average differences in taste perception across the life course on bitter beverage consumption. Thus, if there were a sensitive period for the impact (e.g. if bitter taste perception in early childhood determined drinking habits that persisted into later life) our results would be an underestimate of that effect. Additionally, the use of per SD change in taste perception allows us to assess the effect of changing from perception scale of strong/moderate to moderate/weak, without worrying about within-individual variation in taste perception as observed in earlier test-retest validations^52^. The use of a mixed model approach also allows us to leverage the relatedness in the UKB cohort to gain higher statistical power, resulting in slightly narrower confidence intervals than conventional regression models that exclude related individuals (Supplementary Table 11). Although our choice of a cut-off for high coffee and tea drinkers is arbitrary (based on a percentile), adopting alternative cut-offs made no meaningful difference to the results (Supplementary Table 12).

A major limitation for our study is the relatively small sample size (n = 1757) used to estimate the SNP-bitter taste associations. However, this limitation is unlikely to have substantially biased our finding as all of the SNPs used in this study have been replicated in other independent GWAS^26,35^. As our experiments only consist of one genetic instrument per trait, we were unable to perform sensitivity analyses that explore the validity of IVs through multiple SNPs-based approaches (e.g. MR-Egger^40^ and weighted median^53^ analyses) to evaluate potential bias due to horizontal pleiotropy and therefore we could not exclude the possibility that the SNPs are associated with bitter beverage intake via independent pathways. However, given the functional role of these SNPs in bitter taste receptor genes^27^ and the evidence showing the association of the expression of bitter taste receptor (i.e. TAS2R38) with bitter taste perception and intake of caffeine^54^, these SNPs are more likely to act on the intake of bitter beverage via taste perception. Applying multiple instruments for each trait can potentially allow better assessment of pleiotropy through the use of statistical models, although this will unlikely translate to better precision on our causal estimates given our individual SNP instruments already explain reasonably high amount of variance on bitter tastes. Additionally, our pleiotropic tests using publicly available GWAS summary results did not show evidence of effect of these SNPs on potential confounders (Supplementary Tables 13 and 14). Nevertheless, further research is still required to validate the causal effects detected in the present study.

The response amongst those invited to UK Biobank was ~5% and participants differ considerably from the underlying population with respect to socioeconomic position, health and survival^55^. This could potentially introduce some level of selection bias influencing our MR associations^56^. For example, if non-responsiveness is related to genetic predictors of a number of mental health and lifestyle/behavioural outcomes which happen to also confound drinking behaviour^57^. However, sensitivity analyses (Supplementary Table 13) evaluating the association between these bitter taste SNPs and proxies of socio-economic status (i.e. UK Biobank townsend deprivation index and number of vehicle in household) found no evidence of association. It is possible that behavioural and disease traits correlated to coffee consumption (e.g. diet behaviour, smoking, anorexia, and insomnia) influence bitter taste perception. But using publicly available GWAS summary data^42,43^ we found no strong evidence for this (Supplementary Table 14).

The use of the wald-type estimator to evaluate the causal effect has an intrinsic assumption that the exposure-outcome relationship is linear. For behavioural traits like coffee consumption, this assumption can be violated when we assess the effect of bitter taste on individuals at the extreme end of the trait distribution (i.e. non-tasters, or extremely sensitive tasters). We examined associations both as continuous and binary traits, in general the inference drawn for the continuous measure (e.g. cups per day) was similar to those estimated on drinker status.

Finally, beverage consumption data in the UK Biobank were based on self-reporting which will inevitably contain recall bias and measurement error, but these are unlikely to have inflated Type I error rates in MR analyses. Our findings were based on the intake of black and green tea, and the effects may differ for other tea types with different taste profiles. It is also important to note that our findings do not necessarily generalise to non-European populations.

Overall, our findings demonstrate that differences in bitter taste perception are causally associated with specific bitter beverage consumption behaviour. The magnitudes of causal effects that we see for coffee and tea are small (i.e. 0.02 to 0.15 cups per day for a SD greater bitter taste perception). Given the popularity of these bitter beverages at a population level their consumption could have significant impact on health outcomes, but this requires further investigation.

## Electronic supplementary material

Supplementary materials
