# Author Correction: Understanding the role of bitter taste perception in coffee, tea and alcohol consumption through Mendelian randomization

**DOI:** 10.1038/s41598-020-60488-3

**Published:** 2020-03-11

**Authors:** Jue-Sheng Ong, Liang-Dar Hwang, Victor W. Zhong, Jiyuan An, Puya Gharahkhani, Paul A. S. Breslin, Margaret J. Wright, Deborah A. Lawlor, John Whitfield, Stuart MacGregor, Nicholas G. Martin, Marilyn C. Corneli

**Affiliations:** 1 QIMR Berghofer Medical Research Institute, Brisbane, Australia; 2 School of Medicine, University of Queensland, Brisbane, Australia; 3 University of Queensland Diamantina Institute, University of Queensland, Brisbane, Australia; 4 Department of Preventive Medicine, Northwestern University Feinberg School of Medicine, Chicago, IL USA; 5 Monell Chemical Senses Center, Philadelphia, PA 19104 USA; 6 Department of Nutritional Sciences, School of Environmental and Biological Sciences, Rutgers University, New Brunswick, NJ 08901 USA; 7 Queensland Brain Institute, University of Queensland, Brisbane, Queensland 4072 Australia; 8 Centre for Advanced Imaging, University of Queensland, Brisbane, Queensland 4072 Australia; 9 MRC Integrative Epidemiology Unit at the University of Bristol, Oakfield House, Oakfield Grove, Bristol, BS8 2BN UK; 10 Population Health Science, Bristol Medical School, University of Bristol, Canynge Hall, Whiteladies Road, Bristol, BS8 2PS UK

Correction to: *Scientific Reports* 10.1038/s41598-018-34713-z, published online 15 November 2018

The original version of this Article contained an error in the author name Liang-Dar Hwang, which was incorrectly given as Daniel Liang-Dar Hwang.

This has now been corrected in the PDF and HTML versions of the Article, and in the accompanying Supplementary Information file.

