# Supplementary material for: Understanding the role of bitter taste perception in coffee, tea and alcohol consumption through Mendelian randomization
Source: Sci Rep. 2018 Nov 15;8:16414. doi: 10.1038/s41598-018-34713-z (PMC6237869; doi:10.1038/s41598-018-34713-z)
Supplement: Supplementary file 1 — Supplementary materials [file 41598_2018_34713_MOESM1_ESM.docx]

Supplementary material for manuscript titled

**“Understanding the role of bitter taste perception in coffee, tea and alcohol consumption through Mendelian randomization”**

**Jue-Sheng Ong***^1,2^, Liang-Dar Hwang*^1,2,3^, Victor W. Zhong^4^, Jiyuan An^1^, Puya Gharahkhani^1^, Paul A. S. Breslin^5,6^, Margaret J. Wright^7,8^, Deborah A. Lawlor^9,10^, John Whitfield^1^, Stuart MacGregor^1^, Nicholas G. Martin^1^ and Marilyn C. Cornelis^4^

^1^ QIMR Berghofer Medical Research Institute, Brisbane, Australia.

^2^ School of Medicine, University of Queensland, Brisbane, Australia.

^3^ University of Queensland Diamantina Institute, University of Queensland, Brisbane, Australia.

^4^ Department of Preventive Medicine, Northwestern University Feinberg School of Medicine, Chicago, IL, USA.

^5^ Monell Chemical Senses Center, Philadelphia, PA 19104, USA.

^6^ Department of Nutritional Sciences, School of Environmental and Biological Sciences, Rutgers University, New Brunswick, NJ 08901, USA.

^7^ Queensland Brain Institute, University of Queensland, Brisbane, Queensland 4072, Australia

^8^ Centre for Advanced Imaging, University of Queensland, Brisbane, Queensland 4072, Australia

^9^ MRC Integrative Epidemiology Unit at the University of Bristol, UK.

^10^ Population Health Science, Bristol Medical School, University of Bristol, UK.

* Equal contribution

Correspondence:
**Jue-Sheng Ong**. Email: [juesheng.ong@qimrberghofer.edu.au](mailto:juesheng.ong@qimrberghofer.edu.au)
QIMR Berghofer Medical Research Institute, 300 Herston Road, QLD 4006, Brisbane, Australia.

Table of content

[Supplementary Table 1. Genetic instruments of perceived intensity for PROP, quinine, and caffeine. 3](#_Toc525135286)

[Supplementary Table 2. Frequencies of bitter perception SNP genotypes in UK Biobank samples (N=438,870) 3](#_Toc525135287)

[Supplementary Table 3. Power calculation for mendelian randomization analyses. 4](#_Toc525135288)

[Supplementary table 4. SNP associations with the intake of coffee, tea, and alcohol (linear). 6](#_Toc525135289)

[Supplementary Table 5. SNP associations with the drinker status of coffee, tea, and alcohol (logistic). 6](#_Toc525135290)

[Supplementary Table 6. Cross-conditional MR analyses to evaluate association between increased bitter perception on coffee/tea intake 7](#_Toc525135291)

[Supplementary Table 7. MR association for increased bitter perception with coffee intake among tea non-drinkers 7](#_Toc525135292)

[Supplementary Table 8. MR association for increased bitter perception with tea intake among non-coffee drinkers 8](#_Toc525135293)

[Supplementary Table 9. Stratified analyses on MR association of PROP perception on different types of wine. 9](#_Toc525135294)

[Supplementary Table 10. MR association of bitter taste and bitter beverage consumption quantity stratified by sex. 10](#_Toc525135295)

[Supplementary Table 11. MR association between bitter taste perception and drinker status estimated using conventional logistic regression* 11](#_Toc525135296)

[Supplementary Table 12. Sensitivity analysis evaluating MR association between bitter taste perception and drinker status at different cut-offs for classification of heavy drinkers. 11](#_Toc525135297)

[Supplementary Table 13. Association of bitter perception SNP with potential confounders on beverage consumption (GENEATLAS). 12](#_Toc525135298)

[Supplementary Table 14. Association of bitter perception SNP with potential confounders on beverage consumption (PhenoScanner). 14](#_Toc525135299)

# Supplementary Table 1. Genetic instruments of perceived intensity for PROP, quinine, and caffeine.

| **Trait** | **SNP** | **Chr** | **EA*** | **Beta** | **SE** | $\boldsymbol{r}^{\boldsymbol{2}}$ | **F-statistics** | **Pval** |
| --- | --- | --- | --- | --- | --- | --- | --- | --- |
| PROP | rs1726866 | 7 | G | 9.65E-01 | 2.81E-02 | 45.94% | 901.1 | 5.62E-198 |
| Quinine | rs10772420 | 12 | A | 3.37E-01 | 3.38E-02 | 5.67% | 96.8 | 7.84E-23 |
| Caffeine | rs2597979 | 12 | G | 2.64E-01 | 4.80E-02 | 1.91% | 30.0 | 4.17E-08 |

*EA is the effect allele corresponding to increasing level of perceived intensity.
$\boldsymbol{r}^{\boldsymbol{2}}$ refers to the proportion of phenotypic variance explained by the SNP. Association estimates were extracted from Hwang et al. (submitted).

# Supplementary Table 2. Frequencies of bitter perception SNP genotypes in UK Biobank samples (N=438,870)

| **SNP** | **Ref. Allele (A1)** | **Alt. Allele (A2)** | **Ref Allele Freq.** | **Genotype Freq. (A1A1, A1A2, A2A2)** |
| --- | --- | --- | --- | --- |
| **rs1726866** | G | A | 0.45 | 0.21, 0.49, 0.30 |
| **rs10772420** | G | A | 0.48 | 0.23, 0.50, 0.27 |
| **rs2597979*** | G | C | 0.21 | 0.05, 0.32, 0.63 |

*The genotypes for rs2597979 were imputed using the HRC reference panel. Genotype values were given in dosages and rounded to the nearest integer in calculating percentage of allele carriers.

# Supplementary Table 3. Power calculation for mendelian randomization analyses.

Panel (a) for continuous outcome; (b) for binary outcome. K refers to the sample prevalence of cases (cases/total sample size). Power calculations performed using mRnd web-interface (http://cnsgenomics.com/shiny/mRnd/)

a)

| N | true beta | R^2 | var(exposure) | var(outcome) | Power |
| --- | --- | --- | --- | --- | --- |
| 400000 | 0.05 | 0.4 | 1 | 4 | 0.99 |
| 400000 | 0.03 | 0.4 | 1 | 4 | 0.99 |
| 400000 | 0.05 | 0.08 | 1 | 4 | 0.99 |
| 400000 | 0.04 | 0.08 | 1 | 4 | 0.95 |
| 400000 | 0.03 | 0.08 | 1 | 4 | 0.77 |
| 400000 | 0.075 | 0.02 | 1 | 4 | 0.92 |
| 400000 | 0.05 | 0.02 | 1 | 4 | 0.92 |
| 400000 | 0.04 | 0.02 | 1 | 4 | 0.61 |

b)

| N | K | R^2 | true OR | Power |
| --- | --- | --- | --- | --- |
| 200000 | 0.5 | 0.2 | 1.1 | 0.99 |
| 200000 | 0.5 | 0.02 | 1.1 | 0.85 |
| 150000 | 0.5 | 0.2 | 1.1 | 0.99 |
| 150000 | 0.3 | 0.2 | 1.1 | 0.99 |
| 150000 | 0.5 | 0.08 | 1.2 | 0.99 |
| 150000 | 0.3 | 0.08 | 1.2 | 0.99 |
| 150000 | 0.5 | 0.08 | 1.1 | 0.99 |
| 150000 | 0.3 | 0.08 | 1.1 | 0.99 |
| 150000 | 0.5 | 0.02 | 1.2 | 0.99 |
| 150000 | 0.3 | 0.02 | 1.2 | 0.99 |
| 150000 | 0.5 | 0.02 | 1.1 | 0.74 |
| 150000 | 0.3 | 0.02 | 1.1 | 0.68 |

# Supplementary table 4. SNP associations with the intake of coffee, tea, and alcohol (linear).

|  |  | **Coffee** | | |  | **Tea** | | |  | **Alcohol** | | |
| --- | --- | --- | --- | --- | --- | --- | --- | --- | --- | --- | --- | --- |
| **SNP** | **EA** | **Beta** | **SE** | **Pval** |  | **Beta** | **SE** | **Pval** |  | **Beta** | **SE** | **Pval** |
| rs1726866 | G | -2.00E-02 | 4.70E-03 | 2.20E-05 |  | 3.45E-02 | 6.26E-03 | 3.60E-08 |  | -1.36E-01 | 2.34E-02 | 6.00E-09 |
| rs10772420 | A | -2.74E-02 | 4.69E-03 | 5.00E-09 |  | 2.76E-02 | 6.24E-03 | 9.80E-06 |  | -4.46E-02 | 2.34E-02 | 5.70E-02 |
| rs2597979 | G | 3.86E-02 | 5.93E-03 | 7.70E-11 |  | -4.55E-02 | 7.90E-03 | 8.70E-09 |  | -4.65E-02 | 2.96E-02 | 1.20E-01 |

*EA is the effect allele corresponding to increasing level of perceived intensity.

# Supplementary Table 5. SNP associations with the drinker status of coffee, tea, and alcohol (logistic).

|  |  | **Coffee** | | |  | **Tea** | | |  | **Alcohol** | | |
| --- | --- | --- | --- | --- | --- | --- | --- | --- | --- | --- | --- | --- |
| **SNP** | **EA*** | **log(OR)** | **SE** | **Pval** |  | **log(OR)** | **SE** | **Pval** |  | **log(OR)** | **SE** | **Pval** |
| rs1726866 | G | -3.07E-02 | 7.04E-03 | 1.30E-05 |  | 3.52E-02 | 6.48E-03 | 5.30E-08 |  | -2.99E-01 | 6.89E-02 | 1.10E-05 |
| rs10772420 | A | -3.77E-02 | 7.04E-03 | 8.90E-08 |  | 2.54E-02 | 6.47E-03 | 6.30E-05 |  | 3.37E-01 | 6.88E-02 | 7.70E-01 |
| rs2597979 | G | 4.69E-02 | 8.93E-03 | 1.10E-07 |  | -4.42E-02 | 8.17E-03 | 5.10E-08 |  | 1.89E-02 | 8.72E-02 | 8.50E-01 |

*EA is the effect allele corresponding to increasing level of perceived intensity.
The SNP-outcome association were approximated using log(OR) = beta_bolt/(mu*(1-mu)) where beta_bolt is the slope of the bolt-lmm linear mixed model regression (assuming outcome being continuous) and mu is the sample prevalence (mu=cases/(cases + controls)).

# Supplementary Table 6. Cross-conditional MR analyses to evaluate association between increased bitter perception on coffee/tea intake

| **Taste** | **1-SD increase in Bitter against coffee adjusted for tea intake** | | | **1-SD increase in Bitter against tea adjusted for coffee intake** | | |
| --- | --- | --- | --- | --- | --- | --- |
|  |  |  |  |  |  |  |
|  | Beta_cond | se_cond | pval | Beta_cond | se_cond | pval |
| PROP | -0.0123 | 0.005 | 1.22E-02 | 0.0277 | 0.0065 | 3.47E-05 |
| Quinine | -0.0677 | 0.014 | 1.27E-06 | 0.047 | 0.019 | 1.34E-02 |
| Caffeine | 0.11 | 0.0225 | 1.07E-06 | -0.124 | 0.031 | 5.88E-05 |

# Supplementary Table 7. MR association for increased bitter perception with coffee intake among tea non-drinkers

| Taste | Unstratified  (n= 408,191) | | |  | Among tea non-drinkers (tea per day <2)  (n=90,706) | | |  | Among strict tea non-drinkers (tea per day <1)  (n=58,951) | | |
| --- | --- | --- | --- | --- | --- | --- | --- | --- | --- | --- | --- |
|  | beta | se | pvalue |  | beta | se | pvalue |  | beta | se | pvalue |
| PROP | -2.00E-02 | 4.70E-03 | 2.20E-05 |  | -0.016 | 0.013 | 0.211 |  | -0.009 | 0.017 | 0.590 |
| Quinine | -2.74E-02 | 4.69E-03 | 5.00E-09 |  | -0.115 | 0.036 | 0.001 |  | -0.124 | 0.048 | 0.010 |
| Caffeine | 3.86E-02 | 5.93E-03 | 7.70E-11 |  | 0.124 | 0.057 | 0.030 |  | 0.130 | 0.077 | 0.090 |

*Unstratified model were performed using BOLT-LMM to account for genetic relatedness among individuals to maximise power. Stratified analyses were performed after removing related individuals.

# Supplementary Table 8. MR association for increased bitter perception with tea intake among non-coffee drinkers

| Taste | Unstratified*  (N = 425,378) | | |  | Among coffee non-drinkers (coffee per day <2)  (N = 165,751) | | |  | Among strict coffee non-drinkers (coffee per day <1)  (N = 85,077) | | |
| --- | --- | --- | --- | --- | --- | --- | --- | --- | --- | --- | --- |
|  | beta | se | pvalue |  | beta | se | pvalue |  | beta | se | pvalue |
| PROP | 3.45E-02 | 6.26E-03 | 3.60E-08 |  | 0.021 | 0.011 | 0.052 |  | 0.021 | 0.017 | 0.208 |
| Quinine | 2.76E-02 | 6.24E-03 | 9.80E-06 |  | 0.043 | 0.031 | 0.167 |  | 0.033 | 0.048 | 0.498 |
| Caffeine | -4.55E-02 | 7.90E-03 | 8.70E-09 |  | -0.157 | 0.050 | 0.002 |  | -0.159 | 0.078 | 0.042 |

*Unstratified model were performed using BOLT-LMM to account for genetic relatedness among individuals to maximise power. Stratified analyses were performed manually in statistical package R after removing related individuals.

# Supplementary Table 9. Stratified analyses on MR association of PROP perception on different types of wine.

| Consumption | beta | se | pval |
| --- | --- | --- | --- |
| All alcohol (frequency score) | 0.01988248705 | 0.0030975 | 5.90E-10 |
| Champagne wine (glass/day) | -0.03915108808 | 0.0117846 | 1.30E-03 |
| Red wine (glass/day) | 0.08847958549 | 0.0140698 | 1.30E-09 |

Note: Association estimates are scaled to 1-SD increase in PROP perception. Alcohol(frequence score) is derived from UK Biobank self-reported alcohol consumption questionnaire (UKB field-ID 1558).

# Supplementary Table 10. MR association of bitter taste and bitter beverage consumption quantity stratified by sex.

| **Bitter taste** | **Beverage** | **Sex-specific causal estimate of increased perception on consumption behaviour** | | | | | |  | **chisq-diff** | **pval of diff.** |
| --- | --- | --- | --- | --- | --- | --- | --- | --- | --- | --- |
|  |  | **Males** | | | **Females** | | |  |  |  |
|  |  | **beta** | **se** | **pval** | **beta** | **se** | **pval** |  |  |  |
| PROP | Coffee | -0.021 | 0.008 | 8.33E-03 | -0.020 | 0.007 | 2.15E-03 |  | 0.007 | 0.932 |
| Quinine |  | -0.107 | 0.023 | 2.17E-06 | -0.073 | 0.019 | 8.48E-05 |  | 1.318 | 0.251 |
| Caffeine |  | 0.149 | 0.037 | 4.76E-05 | 0.154 | 0.030 | 3.22E-07 |  | 0.012 | 0.912 |
|  |  |  |  |  |  |  |  |  |  |  |
| PROP | Tea | 0.047 | 0.010 | 7.97E-06 | 0.024 | 0.009 | 7.48E-03 |  | 2.843 | 0.092 |
| Quinine |  | 0.073 | 0.030 | 1.47E-02 | 0.095 | 0.025 | 1.78E-04 |  | 0.315 | 0.574 |
| Caffeine |  | -0.091 | 0.048 | 6.03E-02 | -0.261 | 0.041 | 1.37E-10 |  | 7.334 | 0.007 |
|  |  |  |  |  |  |  |  |  |  |  |
| PROP | Alcohol | 0.180 | 0.037 | 9.15E-07 | 0.109 | 0.031 | 4.80E-04 |  | 2.215 | 0.137 |
| Quinine |  | 0.003 | 0.105 | 9.78E-01 | -0.076 | 0.031 | 1.50E-02 |  | 0.518 | 0.472 |
| Caffeine |  | 0.029 | 0.169 | 8.62E-01 | 0.081 | 0.039 | 3.80E-02 |  | 0.088 | 0.767 |

# Supplementary Table 11. MR association between bitter taste perception and drinker status estimated using conventional logistic regression*

|  |  | **Coffee** | | |  | **Tea** | | |  | **Alcohol** | | |
| --- | --- | --- | --- | --- | --- | --- | --- | --- | --- | --- | --- | --- |
| **SNP** | **EA** | **log(OR)** | **SE** | **Pval** |  | **log(OR)** | **SE** | **Pval** |  | **log(OR)** | **SE** | **Pval** |
| rs1726866 | G | -2.96E-02 | 7.45E-03 | 7.01E-05 |  | 3.77E-02 | 6.88E-03 | 4.36E-08 |  | -4.12E-02 | 9.28E-03 | 9.18E-06 |
| rs10772420 | A | -4.37E-02 | 7.44E-03 | 4.19E-09 |  | 2.80E-02 | 6.87E-03 | 4.69E-05 |  | -5.53E-03 | 2.66E-02 | 8.35E-01 |
| rs2597979 | G | 4.97E-02 | 9.36E-03 | 1.13E-07 |  | -4.69E-02 | 8.68E-03 | 6.47E-08 |  | 9.69E-03 | 4.30E-02 | 8.22E-01 |

*Related individuals were excluded for these analyses. Model was also adjusted for top 10 ancestral principal components, age and sex.

# Supplementary Table 12. Sensitivity analysis evaluating MR association between bitter taste perception and drinker status at different cut-offs for classification of heavy drinkers.

| **Non vs heavy drinker outcome** | **SNP** | **Perception** | **non-drinkers** | **heavy-drinkers** | **OR** | **lower 95% CI** | **upper 95% CI** |
| --- | --- | --- | --- | --- | --- | --- | --- |
| coffee (<1 vs >5 cup/day) | rs10772420 | Quinine | 93406 | 28084 | 0.8970 | 0.8486 | 0.9481 |
| coffee (<1 vs >5 cup/day) | rs2597979 | Caffeine | 93406 | 28084 | 1.2487 | 1.1411 | 1.3664 |
| coffee (<1 vs >5 cup/day) | rs1726866 | PROP | 93406 | 28084 | 0.9657 | 0.9472 | 0.9846 |
| coffee (<2 vs >4 cup/day) | rs10772420 | Quinine | 181719 | 50937 | 0.8941 | 0.8582 | 0.9314 |
| coffee (<2 vs >4 cup/day) | rs2597979 | Caffeine | 181719 | 50937 | 1.1944 | 1.1178 | 1.2763 |
| coffee (<2 vs >4 cup/day) | rs1726866 | PROP | 181719 | 50937 | 0.9687 | 0.9550 | 0.9827 |
| tea (<1 vs >5 cup/day) | rs10772420 | Quinine | 64775 | 87120 | 1.0930 | 1.0478 | 1.1400 |
| tea (<1 vs >5 cup/day) | rs2597979 | Caffeine | 64775 | 87120 | 0.8120 | 0.7586 | 0.8691 |
| tea (<1 vs >5 cup/day) | rs1726866 | PROP | 64775 | 87120 | 1.0393 | 1.0241 | 1.0547 |
| tea (<2 vs >5 cup/day) | rs10772420 | Quinine | 100030 | 87120 | 1.0782 | 1.0384 | 1.1196 |
| tea (<2 vs >5 cup/day) | rs2597979 | Caffeine | 100030 | 87120 | 0.8457 | 0.7960 | 0.8986 |
| tea (<2 vs >5 cup/day) | rs1726866 | PROP | 100030 | 87120 | 1.0372 | 1.0236 | 1.0509 |

# Supplementary Table 13. Association of bitter perception SNP with potential confounders on beverage consumption (GENEATLAS).

| **SNP** | **Perception** | **Effect Allele** | **Potential Confounder** | **Magnitude of association** | **P-value** |
| --- | --- | --- | --- | --- | --- |
|  |  |  |  |  |  |
| Diet |  |  |  |  |  |
| rs1726866 | PROP | A | Cheese intake | -5.83E-03 | 9.35E-03 |
| rs10772420 | Quinine | A | Cheese intake | 4.72E-03 | 3.53E-02 |
| rs10845296 | Caffeine | A | Cheese intake | 8.49E-03 | 2.37E-03 |
| rs1726866 | PROP | A | Variation in diet | 7.99E-04 | 5.35E-01 |
| rs10772420 | Quinine | A | Variation in diet | -1.50E-03 | 2.43E-01 |
| rs10845296 | Caffeine | A | Variation in diet | -4.56E-04 | 7.76E-01 |
|  |  |  |  |  |  |
| Tobacco use |  |  |  |  |  |
| rs1726866 | PROP | A | Smoking status | 6.24E-04 | 6.58E-01 |
| rs10772420 | Quinine | A | Smoking status | 1.89E-04 | 8.93E-01 |
| rs10845296 | Caffeine | A | Smoking status | 7.83E-04 | 6.55E-01 |
| rs1726866 | PROP | A | Current tobacco smoking | 2.69E-04 | 6.78E-01 |
| rs10772420 | Quinine | A | Current tobacco smoking | 4.44E-04 | 4.93E-01 |
| rs10845296 | Caffeine | A | Current tobacco smoking | 3.89E-04 | 6.30E-01 |
|  |  |  |  |  |  |
| Beverage temperature | |  |  |  |  |
| rs1726866 | PROP | A | Hot drink temperature | 5.47E-04 | 6.52E-01 |
| rs10772420 | Quinine | A | Hot drink temperature | 1.48E-03 | 2.21E-01 |
| rs10845296 | Caffeine | A | Hot drink temperature | -1.95E-03 | 1.96E-01 |
|  |  |  |  |  |  |
| Sleep related traits | |  |  |  |  |
| rs1726866 | PROP | A | Sleep duration | -3.77E-03 | 1.07E-01 |
| rs10772420 | Quinine | A | Sleep duration | -2.50E-03 | 2.83E-01 |
| rs10845296 | Caffeine | A | Sleep duration | -3.21E-03 | 2.69E-01 |
| rs1726866 | PROP | A | G47 Sleep disorders | -5.17E-05 | 8.35E-01 |
| rs10772420 | Quinine | A | G47 Sleep disorders | -3.88E-06 | 9.88E-01 |
| rs10845296 | Caffeine | A | G47 Sleep disorders | -2.07E-05 | 9.47E-01 |
| rs1726866 | PROP | A | sleep apnoea | -8.58E-05 | 4.95E-01 |
| rs10772420 | Quinine | A | sleep apnoea | 1.36E-05 | 9.14E-01 |
| rs10845296 | Caffeine | A | sleep apnoea | -2.31E-05 | 8.83E-01 |
|  |  |  |  |  |  |
| Socio-economic | |  |  |  |  |
| rs1726866 | PROP | A | Townsend deprivation index at recruitment | 3.59E-03 | 5.49E-01 |
| rs10772420 | Quinine | A | Townsend deprivation index at recruitment | 6.49E-03 | 2.79E-01 |
| rs10845296 | Caffeine | A | Townsend deprivation index at recruitment | -1.51E-04 | 9.84E-01 |
| rs1726866 | PROP | A | Body mass index (BMI) | -5.78E-03 | 5.35E-01 |
| rs10772420 | Quinine | A | Body mass index (BMI) | -4.11E-04 | 9.65E-01 |
| rs10845296 | Caffeine | A | Body mass index (BMI) | -6.50E-03 | 5.80E-01 |
| rs1726866 | PROP | A | Number of vehicles in household | -0.00328 | 0.070207 |
| rs10772420 | Quinine | A | Number of vehicles in household | -0.00192 | 0.28657 |
| rs10845296 | Caffeine | A | Number of vehicles in household | 0.001155 | 0.60741 |

*Estimates were extracted from the GENEATLAS PheWAS database for UK Biobank traits (available at <http://geneatlas.roslin.ed.ac.uk/>). SNP rs10845296 is used as the best proxy in high-LD (Linkage Disequilibrium) with rs2597979 at an r^2 of 0.83. Effect allele refers to the allele associated with the magnitude of association on the potential confounder for a given SNP.

# Supplementary Table 14. Association of bitter perception SNP with potential confounders on beverage consumption (PhenoScanner).

| **SNP** | **Perception** | **Effect/Ref. Allele** | **Potential Confounder** | **Magnitude of association** | **Pvalue** |
| --- | --- | --- | --- | --- | --- |
| Gastrointestinal disorders | |  |  |  |  |
| rs1726866 | PROP | G/A | Crohns disease | -1.03E-02 | 6.66E-01 |
| rs1726866 | PROP | G/A | Inflammatory bowel disease | -4.00E-03 | 8.22E-01 |
| rs1726866 | PROP | G/A | Ulcerative colitis | 3.00E-04 | 9.91E-01 |
| rs10772420 | Quinine | A/G | Crohns disease | -1.30E-02 | 5.87E-01 |
| rs10772420 | Quinine | A/G | Inflammatory bowel disease | -4.70E-03 | 7.91E-01 |
| rs10772420 | Quinine | A/G | Ulcerative colitis | -2.44E-02 | 2.84E-01 |
| rs2597979 | Caffeine | C/G | Crohns disease | 7.50E-03 | 8.07E-01 |
| rs2597979 | Caffeine | C/G | Inflammatory bowel disease | -2.50E-03 | 9.11E-01 |
| rs2597979 | Caffeine | C/G | Ulcerative colitis | -3.08E-02 | 2.74E-01 |
|  |  |  |  |  |  |
| Smoking |  |  |  |  |  |
| rs1726866 | PROP | G/A | Cigarettes per day | -3.24E-02 | 7.20E-01 |
| rs1726866 | PROP | G/A | Ever smoker | 1.16E-02 | 3.83E-01 |
| rs1726866 | PROP | G/A | Former smoker | -3.63E-02 | 4.47E-02 |
| rs1726866 | PROP | G/A | log(Age of smoking onset) | 2.20E-03 | 4.32E-01 |
| rs10772420 | Quinine | A/G | Cigarettes per day | 4.86E-02 | 5.49E-01 |
| rs10772420 | Quinine | A/G | Ever smoker | 1.37E-02 | 2.31E-01 |
| rs10772420 | Quinine | A/G | Former smoker | -4.33E-02 | 6.09E-03 |
| rs10772420 | Quinine | A/G | log(Age of smoking onset) | -2.30E-03 | 2.97E-01 |
